# Identification of high-copy number long terminal repeat retrotransposons and their expansion in *Phalaenopsis* orchids

**DOI:** 10.1186/s12864-020-07221-6

**Published:** 2020-11-19

**Authors:** Chia-Chi Hsu, Shu-Yun Chen, Pei-Han Lai, Yu-Yun Hsiao, Wen-Chieh Tsai, Zhong-Jian Liu, Mei-Chu Chung, Olivier Panaud, Hong-Hwa Chen

**Affiliations:** 1 Department of Life Sciences, National Cheng Kung University, Tainan, Taiwan; 2 Orchid Research and Development Center, National Cheng Kung University, Tainan, Taiwan; 3 Institute of Tropical Plant Sciences and Microbiology, National Cheng Kung University, Tainan, Taiwan; 4 Key Laboratory of National Forestry and Grassland Administration for Orchid Conservation and Utilization at College of Landscape Architecture, Fujian Agriculture and Forestry University, Fuzhou, China; 5 Institute of Plant and Microbial Biology, Academia Sinica, Nankang, Taipei, Taiwan; 6 Institute of Plant Genome and Development, University of Perpignan, Perpignan, France

**Keywords:** Expression level, Genome size, Gypsy, *Orchid-rt1*, Intron, Orchids, *Phalaenopsis*, Retrotransposon

## Abstract

**Background:**

Transposable elements (TEs) are fragments of DNA that can insert into new chromosomal locations. They represent a great proportion of eukaryotic genomes. The identification and characterization of TEs facilitates understanding the transpositional activity of TEs with their effects on the orchid genome structure.

**Results:**

We combined the draft whole-genome sequences of *Phalaenopsis equestris* with BAC end sequences, Roche 454, and Illumina/Solexa, and identified long terminal repeat (LTR) retrotransposons in these genome sequences by using LTRfinder and classified by using Gepard software. Among the 10 families *Gypsy*-like retrotransposons, three families *Gypsy1*, *Gypsy2*, and *Gypsy3*, contained the most copies among these predicted elements. In addition, six high-copy retrotransposons were identified according to their reads in the sequenced raw data. The 12-kb *Orchid-rt1* contains 18,000 copies representing 220 Mbp of the *P. equestris* genome. Southern blot and slot blot assays showed that these four retrotransposons *Gypsy1*, *Gypsy2*, *Gypsy3*, and *Orchid-rt1* contained high copies in the large-genome-size/large-chromosome species *P. violacea* and *P. bellina*. Both *Orchid-rt1* and *Gypsy1* displayed various ratios of copy number for the LTR sequences versus coding sequences among four *Phalaenopsis* species, including *P. violacea* and *P. bellina* and small-genome-size/small-chromosome *P. equestris* and *P. ahprodite* subsp. *formosana*, which suggests that *Orchid-rt1* and *Gypsy1* have been through various mutations and homologous recombination events. FISH results showed amplification of *Orchid-rt1* in the euchromatin regions among the four *Phalaenopsis* species. The expression levels of *Peq018599* encoding copper transporter 1 is highly upregulated with the insertion of *Orchid-rt1*, while it is down regulated for *Peq009948* and *Peq014239* encoding for a 26S proteasome non-ATP regulatory subunit 4 homolog and auxin-responsive factor AUX/IAA-related. In addition, insertion of *Orchid-rt1* in these three genes are all in their intron regions.

**Conclusion:**

*Orchid-rt1* and *Gypsy1–3* have amplified within *Phalaenopsis* orchids concomitant with the expanded genome sizes, and *Orchid-rt1* and *Gypsy1* may have gone through various mutations and homologous recombination events. Insertion of *Orchid-rt1* is in the introns and affects gene expression levels.

**Supplementary Information:**

The online version contains supplementary material available at 10.1186/s12864-020-07221-6.

## Background

Containing more than 25,000 species, Orchidaceae family, classified in class Liliopsida, order Asparagales, is one of the largest angiosperm families. The genus *Phalaenopsis*, an elegant and popular orchid, comprises approximately 63 species [1]. All diploid species of *Phalaenopsis* have the same chromosome number (2n = 2x = 38) [1], but their DNA contents and karyotypes vary significantly. The C value refers to the nuclear DNA contents of the replicated haploid genome and represents the genome size of species [2]. Flow cytometry revealed that the 2C values range from 2.77 pg for *P. philippinensis* to 17.47 pg for *P. lobbii* within the 50 species of *Phalaenopsis* [3, 4]. Two native species in Taiwan, *P. equestris* and *P. aphrodite* subsp. *formosana* (hereafter called *P. aphrodite*), have relatively small genome sizes, with 2C values of 2.95 and 2.81 pg, equivalent to 1.6- and 1.52-Gbp genome sequences [3, 4]. However, the scented *Phalaenopsis* species *P. violacea* and *P. bellina* have a large genome (Additional file 1: Fig. S1), with 2C values of 12.89 and 13.04 pg, respectively, equivalent to 6.99- and 7.07-Gbp genome sizes.

Karyotype analysis of nine *Phalaenopsis* species revealed the genome size associated with the chromosome size. One group with small and uniform chromosomes (1–2.5 μm) includes *P. aphrodite*, *P. stuartiana*, *P. equestris*, *P. cornu-cervi* and *P. lueddemanniana*, and the other with bimodal karyotypes, with small, medium and large chromosomes, includes *P. venosa*, *P. amboinensis*, *P. violacea* and *P. mannii* [5]. Different chromosome sizes among various species result in incompatability for interspecific crosses [5–7], thus limiting the breeding of new varieties for the orchid industry. The amount of constitutive heterochromatin in the interphase nucleus indicates that *Phalaenopsis* species with large chromosomes contain more heterochromatin and repetitive sequences than those with small chromosomes [5].

Mobile or transposable elements (TEs) are DNA fragments that can “jump” to new chromosomal locations and thus often duplicate themselves. They represent a large part of eukaryotic genomes, ranging from 3% in baker’s yeast (*Saccharomyces cerevisiae*) [8], ~20% in fruit fly (*Drosophila melanogaster*) [9], 45% in humans (*Homo sapiens*) [10], 50% in grape (*Vitis vinifera*), 59% in Sorghum (*Sorghum bicolor*), 60% in Amborella (*Amborella trichopoda*), 62% in *P. equestris*, and 75% in maize (*Zea mays*) [11]. In general, there are two major groups of TEs distinguished by their transposition intermediate: class I retrotransposons with “copy-and-paste” retrotransposition, and class II DNA transposons with “cut-and-paste” retrotransposition [12].

Retrotransposons with the “copy-and-paste” mechanism represent the largest portion of higher plant genomes, and long terminal repeat (LTR) retrotransposons are the most common and ubiquitous in the plant kingdom [13, 14]. LTR retrotransposons are related to retroviruses in terms of their structure and retrotransposition mechanism. The complete copies of retrotransposons consist of two LTRs that flank an internal region containing two genes, *gag* and *pol*. The *gag* gene encodes a capsid-like protein, and the *pol* gene encodes a polyprotein with protease, reverse transcriptase, RNase H, and integrase activities. LTR retrotransposons are further classified into two major families, *Ty1*/*copia*-like and *Ty3*/*gypsy*-like elements, depending on the order of reverse transcriptase and integrase.

LTR sequences are responsible for transcription initiation and termination of the internal genes for retrotransposition, leading to an increase in copy number. In contrast, the increased genome size can be mitigated by unequal homologous recombination, generating a much shorter solo LTR, or by illegitimate recombination, leading to element deletion [15]. Therefore, differential efficiency of such increasing versus decreasing mechanisms among plant genomes could explain at least in part the various genome sizes in recently described plant species [15].

Although LTR retrotransposons comprise the largest fraction of most plant genomes, only a few LTR retrotransposon families successfully represent the bulk of the genome. The main TE groups are ancient and are present in all plant kingdoms, yet TE contents show extreme diversity among species in the classes of TEs present, their fraction in the genome and level of transposition activity. Identifying TEs and investigating their impact on each plant species are essential for understanding their functional roles. However, precise characterization of TEs is not easy or straightforward. First, many TEs are structurally incomplete because of intra- or inter-element unequal recombination or accumulation of small deletions by illegitimate recombination [16, 17]. Second, many TEs are grouped in nested patterns [18] or chimerical structures [19]. Finally, low-copy-number TE families are highly divergent across species, and their identification is difficult by sequence homology because of few numbers of previously characterized elements. Hence, the full characterization of TEs is a critical step for precise gene annotation in a sequenced genome and investigating the effects between TEs and genome evolution.

Although the whole-genome sequences of *Phalaenopsis* have been published for two native Taiwan species, *P. equestris* and *P. aphrodite* [11, 20], few studies have focused on the TEs contributing to their genome structure and evolution. Recently, a *P instability factor*-like transposon, *PePIF1,* was isolated from *P. equestris* and seemed to be actively transposed during microprogation [21]. A retrotransposon, *Harlequin Orchid RetroTransposon 1* (*HORT1*), is inserted in the upstream regulatory sequence of an anthocyanin-related *PeMYB11* and results in its high expression and the harlequin/black flowers of *Phalaenopsis* orchids [22].

In this study, we have identified four high-copy retrotransposons, and further examined in both small-genome-size/small-chromosome *Phalaenopsis* species (*P. aphrodite* and *P. equestris*) and large-genome-size/large-chromosome species (*P. bellina* and *P. violacea*).

## Results

### Identification of long terminal repeat (LTR) retrotransposons

The LTR retrotransposons were identified from several sequenced genome datasets for *Phalaenopsis* orchids, including BAC end sequences (BESs) [23], Roche 454, Illumina/Solexa, and assembled draft whole genome sequences [11], by using two approaches. First, these sequences were assembled into large contigs, then LTRfinder was used to identify the full-length LTR retrotransposons, which are bordered by two identical LTRs at the time of insertion and subsequently diverged by random mutations [24]. Second, the high-copy numbers of retrotransposons with high similarities were predicted from the sequencing reads, then those with the highest copies in the sequenced raw data were selected.

**Fig. 1:**
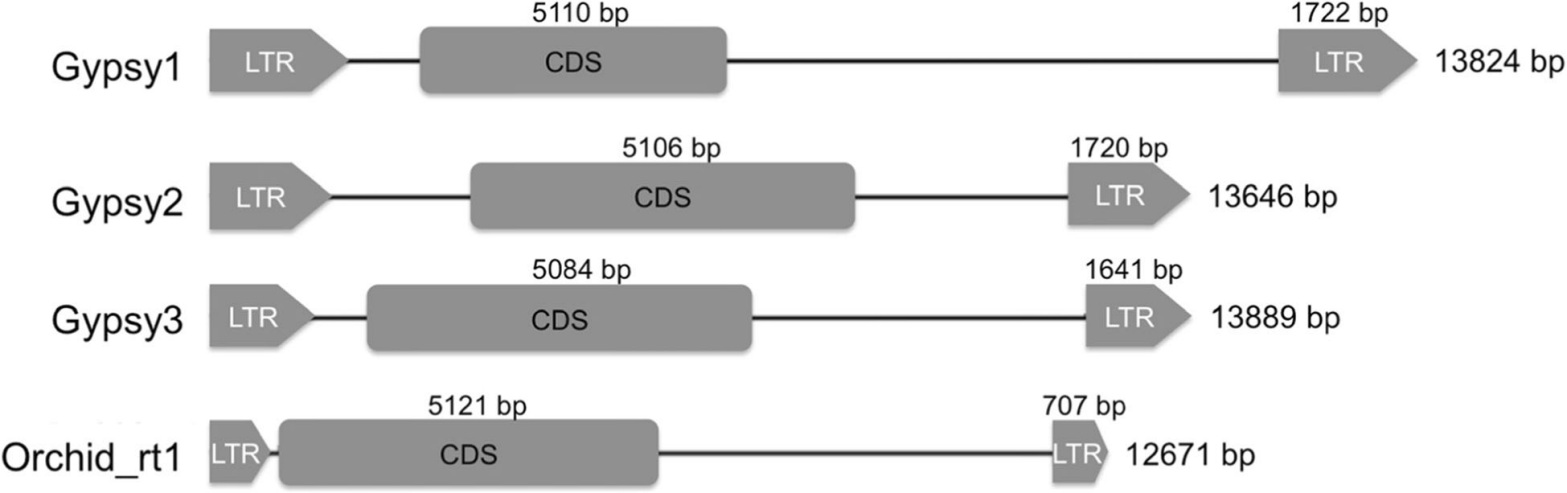
A diagram for the sequence structures of the *Gypsy1* to *Gypsy3* and *Orchid-rt1*. Long terminal repeats (LTRs) and protein-coding sequence (CDS) were shown as arrows and boxes, respectively

In total, 906 *Gypsy*-like and 402 *Copia*-like LTR retrotransposons were identified as the full-length elements by using LTRfinder, then further separated into different families according to their sequence homology by using a Dot-Plot software, Gepard [25]. Overall, 906 *Gypsy*-like retrotransposons were grouped into 10 families (Table 1), and 402 *Copia*-like retrotransposons were distributed into 20 families (Additional file 2: Table S1). Three *Gypsy*-like families, *Gypsy1*-*Gypsy3*, contained the most full-length copies among these predicted elements. The *Gypsy1* family contained 13,824-bp sequences with 1722-bp LTR sequences and a 5110-bp protein-coding region (Table 1, Fig. 1). The *Gypsy2* family had 13,646-bp sequences with 1720-bp LTR sequences and a 5106-bp protein-coding region (Table 1, Fig. 1). The *Gypsy3* family had 13,889-bp sequences with 1641-bp LTR sequences and a 5084-bp protein-coding region (Table 1, Fig. 1). The length of the full element of *Gypsy4 ~ 10* ranged from 8663- to 16,646-bp sequences with 1023- to 2642-bp sequences of LTR sizes. In contrast, the *Copia*-like retrotransposons contained lower copy numbers than those of *Gypsy*, with the highest copy number of 48 in 402 elements (Additional file 2: Table S1).

**Table 1:** The copy numbers of the predicted *Gypsy*-like retrotransposons

Family name	No. of elements	Total length (bp)	LTR length	CDS length (bp)
*Gypsy1*	234	13,824	1722	5110
*Gypsy2*	201	13,646	1720	5106
*Gypsy3*	185	13,889	1641	5084
*Gypsy4*	47	13,180	1629	3356
*Gypsy5*	39	14,447	1872	5099
*Gypsy6*	28	15,199	1949	4880
*Gypsy7*	20	15,648	1813	3145
*Gypsy8*	13	8663	1023	3346
*Gypsy9*	11	16,646	2642	3453
*Gypsy10*	3	15,195	1526	3212
Singleton	125			
total	906

Considering that the new amplified retrotransposons with very similar sequence would be assembled into the same contig after next-generation sequencing and could not be identified as a high-copy element by LTRfinder and Gepard, we used a second approach of high-copy retrotransposons with high similarity predicted as the most sequenced reads in the sequenced raw data. In total, six high-copy number retrotransposons were identified, including one 16S ribosomal DNA (*Orchid-its16S*), one miniature inverted-repeat TE (mite)-type retrotransposon (*Orchid-mite1*), one anc-like retrotransposon (*Orchid-rt-anc1*), and three retrotransposons (*Orchid-rt1*, *Orchid-rt2*, and *Orchid-rt3*) (Table 2). Among them, *Orchid-rt1* is a *Gypsy*-like retrotransposon and contains 12,671-bp sequences with 707-bp LTR sequences and a 5121-bp protein-coding region (Fig. 1). Based on the numbers of these sequenced reads, *Orchid-rt1* is predicted to be present with 18,000 copies in the *P. equestris* genome, accounting for 220-Mbp sequences and 13.8% of the *Phalaenopsis* genome.

**Table 2:** The copy numbers of the predicted high-copy orchid retrotransposons

element	size	454	BES	Illimina	% 454	% BES	% Illumina
*Orchid-its16S*	2060	1625	345	20,914	0.004	0.019	0.006
*Orchid-mite1*	317	500	30	664	0.001	0.002	0.000
*Orchid-rt-anc1*	3396	6961	764	59,493	0.016	0.041	0.018
*Orchid-rt1*	12,754	57,245	2927	358,553	0.130	0.158	0.107
*Orchid-rt2*	3059	4650	247	41,109	0.011	0.013	0.012
*Orchid-rt3*	3410	17,457	1999	112,842	0.040	0.108	0.034
Total		439,604	18,486	3,354,752			

### Slot blot assay of high copy number of retrotransposons present in *Phalaenopsis* genomes

Because the repetitive sequences represent a large proportion of the plant genomes and play roles in genomic diversification and speciation [26–28], a rapid burst of retrotransposition could induce genome size changes with different evolutionary effects in the same genus, as reported for *Oryza*, *Nicotiana*, and *Genlisea* [29]. To trace the retrotransposition events of the four high-copy LTR retrotransposons (*Gypsy1*, *Gypsy2*, *Gypsy3*, and *Orchid-rt1*) in four *Phalaenopsis* species, slot blot analysis was used to estimate the copy number of these high-copy retrotransposons with the probes corresponding to the individual LTR region. Four *Phalaenopsis* genomes were analyzed including *P. equestris* and *P. aphrodite* with small chromosomes, and *P. violacea* and *P. bellina* with both large and small chromosomes [5].

**Fig. 2:**
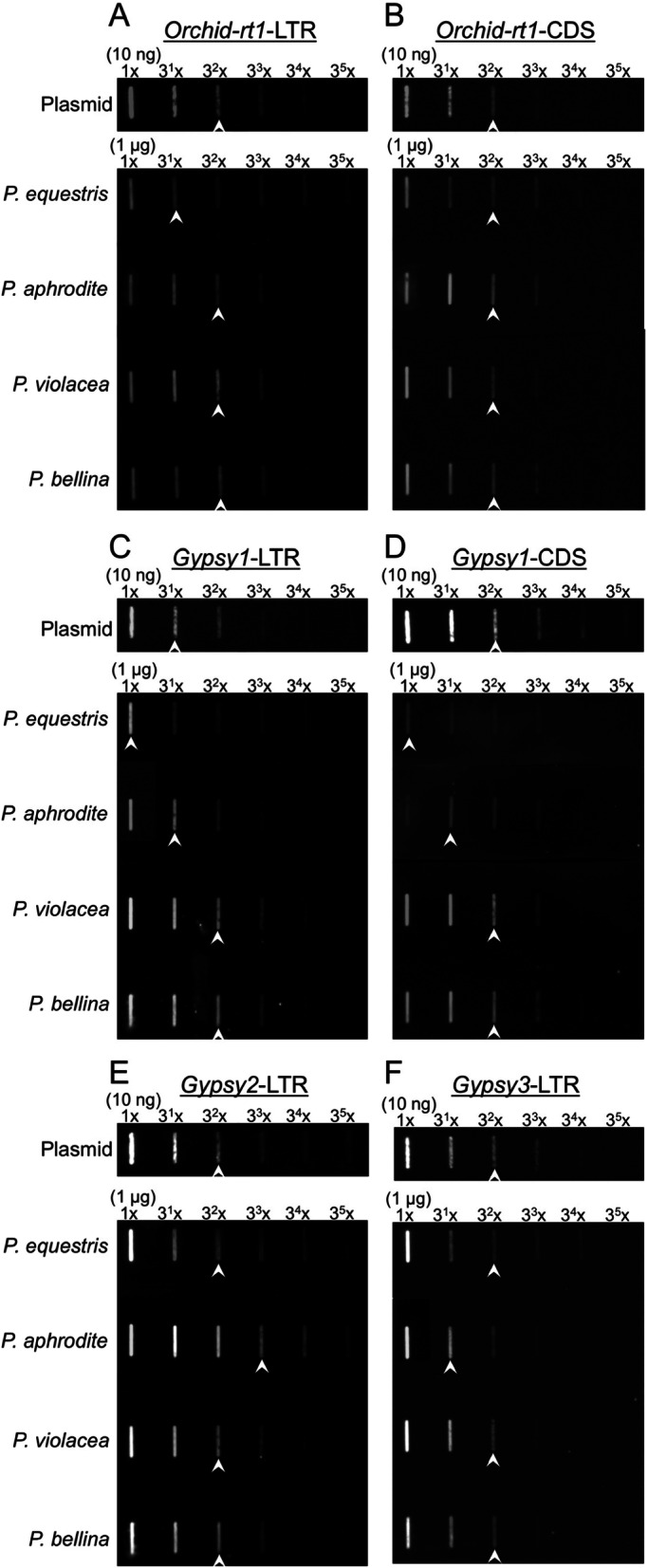
Slot Blot showed the percentage of the LTR (**a**, **c**) and CDS (**b**, **d**) sequences of *Orchid-rt1* (**a**, **b**) and *Gypsy1* (**c**, **d**), and the LTR sequences of *Gypsy2* (**e**) and *Gypsy3* (**f**) within the genomic DNA of *P. equestris, P. aphrodite, P. violacea, and P. belllina.* A 10 ng of plasmid DNA containing the LTR sequences and 1 μg genomic DNA were performed for serial dilution and hybridized with the probes of *PeAct9* and these LTR sequences. Arrowheads indicate the similar intensity of hybridized slots between the plasmid DNA and genomic DNA

A 10-ng plasmid DNA, containing a 707-bp LTR sequence of *Orchid-rt1* and a 3015-bp sequence of T-easy vector, was used as a standard containing the *Orchid-rt1*-LTR sequence for 1.9 ng (Fig. 2). The slot blot results showed that 3^−2^x diluted 10-ng *Orchid-rt1*-LTR plasmid DNA revealed a similar intensity of hybridized spots as 3^−1^x diluted genomic DNA of *P. equestris* (Fig. 2a, arrowheads), which suggests an approximately 0.63-ng (1.9 ng ÷ 3) LTR sequence for *Orchid-rt1* within 1 μg genomic DNA of *P. equestris*. With the genome size of *P. equestris* of 1.6 × 10^9^ bp [11], the LTR sequence for *Orchid-rt1* was 1.01 × 10^6^ bp, for about 1426 copies within the *P. equestris* genome (Table 3). The genome sizes of *P. equestris*, *P. aphrodite*, *P. violacea*, and *P. bellina* were compared to their nuclear DNA content (Table 3); the estimated copy numbers of LTR sequences for *Orchid-rt1* were 1426, 4085, 18,785, and 19,000 in the genomes of *P. equestris*, *P. aphrodite*, *P. violacea*, and *P. bellina*, respectively (Table 3).

**Table 3:** Estimated copies of four transposable elements among the genomes of four *Phalaenopsis* species

		*P. equestris*	*P. aphrodite*	*P. violacea*	*P. bellina*
Nuclear DNA content 1C value (pg)		2.95	2.81	12.89	13.04
Estimated genome size (10^9^ bp)		1.6	1.52	6.99	7.07
genomic DNA (ng/μl)	*Orchid-rt1*-LTR/CDS	0.63/6.3	1.9/6.3	1.9/6.3	1.9/6.3
	*Gypsy1*-LTR/CDS	1.2/0.7	3.6/2.1	10.8/6.3	10.8/6.3
	*Gypsy2*-LTR	3.6	10.8	3.6	3.6
	*Gypsy3*-LTR	3.5	1.17	3.5	3.5
Mb/genome	*Orchid-rt1*-LTR/CDS	1.008/10.08	2.888/9.576	13.281/44.037	13.433/44.541
	*Gypsy1*-LTR/CDS	1.92/1.12	5.472/3.192	75.492/44.037	76.356/44.541
	*Gypsy2*-LTR	5.76	16.416	25.164	25.452
	*Gypsy3*-LTR	5.6	1.778	24.465	24.745
Copy no. /genome	*Orchid-rt1*-LTR/CDS	1426/1968	4085/1870	18,785/8599	19,000/8698
	*Gypsy1*-LTR/CDS	1115/219	3178/625	43,840/8618	44,341/8716
	*Gypsy2*-LTR	3349	9544	14,630	14,798
	*Gypsy3*-LTR	3413	1083	14,909	15,079

Similarly, a 10-ng plasmid DNA, having a 5121-bp protein-coding sequence (CDS) of *Orchid-rt1* and a 3015-bp sequence of T-easy vector, was used as a standard with the *Orchid-rt1*-CDS of 6.3 ng (Fig. 2). Slot blot results showed that 3^−3^x diluted 10-ng *Orchid-rt1*-CDS plasmid DNA revealed a similar intensity of hybridized spots as 3^−3^x diluted genomic DNA of *P. equestris* (Fig. 2b, arrowheads), which suggests an approximate 6.3-ng CDS for *Orchid-rt1* within 1 μg genomic DNA of *P. equestris*. So the CDS for *Orchid-rt1* was 10.08 × 10^6^ bp, for about 1968 copies in the *P. equestris* genome (Table 3), and the estimated copy numbers of the CDS for *Orchid-rt1* were 1870, 8599, and 8698 within the genomes of *P. aphrodite*, *P. violacea*, and *P. bellina*, respectively (Table 3).

In comparing the copy numbers of LTR and CDS for *Orchid-rt1*, we found nearly 2-fold copy numbers of LTR versus CDS in the genomes of *P. aphrodite*, *P. violacea*, and *P. bellina*, so most of *Orchid-rt1* involved full-length copies flanked by two LTR sequences in these genomes*.* However, *P. equestris* showed lower copies of LTR than CDS for *Orchid-rt1,* with 1426 and 1968 copies, respectively (Table 3). In the “increase/decrease model”, whereby following the retrotransposon amplification, these sequences without selection pressure are eliminated by homologous or illegitimate recombination and lead to the solo-LTR formation and deletion [15], the copies of *Orchid-rt1* in *P. equestris* were amplified a long time ago and underwent faster TE turnover rate than that in *P. aphrodite*, *P. violacea*, and *P. bellina*.

### Increased copy number of *Gypsy1* coincident with large-genome-size/large-chromosome *Phalaenopsis* species

The copy numbers of LTR and CDS for *Gypsy1* and the LTR for *Gypsy2* and *Gypsy3* were estimated in these four *Phalaenopsis* genomes (Table 3). All retrotransposons contained high copy numbers in the large-chromosome *P. violacea* and *P. bellina* genomes (Table 3), with a much higher copy number of LTR for *Gypsy1* in both *P. violacea* and *P. bellina* genomes, 43,840 and 44,341 copies, respectively (Table 3). Considering that the genome sizes were about 4.5-fold higher for *P. violacea* and *P. bellina* than *P. equestris* and *P. aphrodite*, the copy numbers of LTR and CDS for *Gypsy1* showed about a 39- and 13-fold increase in both *P. violacea* and *P. bellina* as compared with *P. equestris* and *P. aphrodite*, respectively (Table 3), which suggests that *Gypsy1* may be linked to the increased chromosome size as well as genome size in *P. violacea* and *P. bellina*. In addition, *Gypsy1* showed about 5-fold more copies of LTR than CDS in all four *Phalaenopsis* genomes (Table 3), which indicates that *Gypsy1* amplified its copies a long time ago and each copy underwent deletion and recombination events, which resulted in solo-LTR copies [15].

Even though both *P. equestris* and *P. ahprodite* have similar genome size, we found a prevalence for *Gypsy2* or *Gypsy3* in the *P. aphrodite* or *P. equestris* genome. The LTR copy number for *Gypsy2* was higher in *P. aphrodite* than *P. equestris*, but that for *Gypsy3* was higher in *P. equestris* than *P. aphrodite* (Table 3). Therefore, the evolutionary fates among the four retrotransposons varied among *Phalaenopsis* genomes and should be investigated separately.

### Chromosomal localization of *Orchid-rt1* in *Phalaenopsis* genomes

**Fig. 3:**
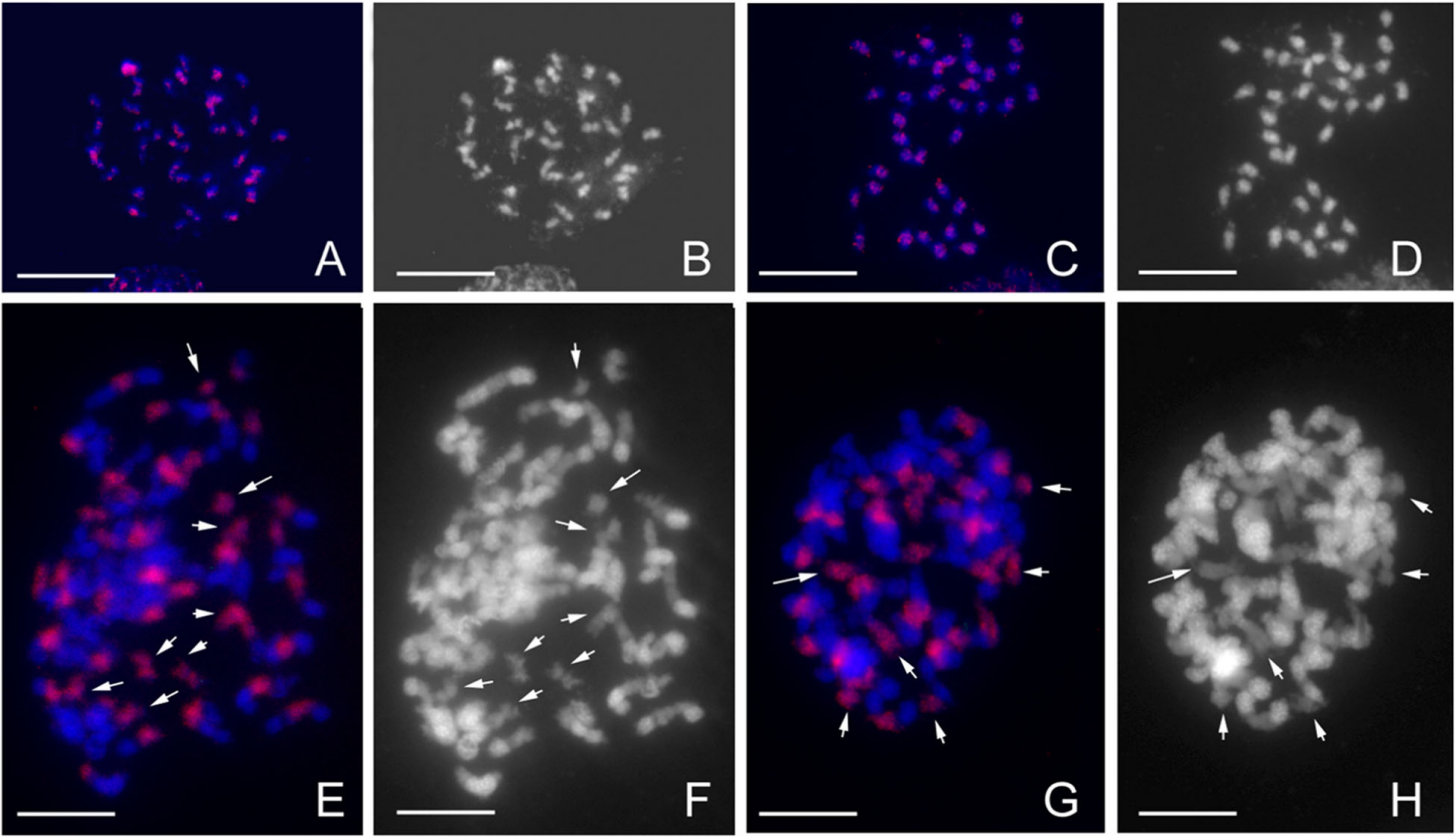
Fluorescence in situ hybridization (FISH) mapped the LTR sequence of *Orchid-rt1* on chromosomes of *P. equestris* (**a**-**b**), *P. aphrodite* subsp. *formosana* (**c**-**d**), *P. bellina* (**e**-**f**), and *P. violacea* (**g**-**h**). **a**, **c**, **e** and **g** The distribution of *Orchid-rt1* (red) on chromosomes (blue). **b**, **d**, **f**, **h** The respective images of chromosomes with DAPI staining. Arrows indicate the small chromosomes in *P. bellina* and *P. violacea*

*Orchid-rt1*, accounting for 13.8% of the *P. equestris* genome size was the highest-copy retrotranspsons and with high similarity among copies. We analyzed its distribution patterns on chromosomes of *P. equestris*, *P. aphrodite*, *P. violacea*, and *P. bellina* by using fluorescence in situ hybridization (FISH). Both *P. equestris* and *P. aphrodite* have small and uniform chromosomes, whereas *P. bellina* and *P. violace* have bimodal karyotypes with small, medium and large chromosomes (Fig. 3). FISH results showed *Orchid-rt1* dispersed through all chromosomes in *P. equestris* (Fig. 3a) and *P. aphrodite* (Fig. 3c). Because the intensity of FISH signals may reflect the abundance of sequences clustered at the regions, *P. bellina* (Fig. 3e) and *P. violacea* (Fig. 3g) have more *Orchid-rt1* as compared with *P. equestris* (Fig. 3a) and *P. aphrodite* (Fig. 3c)*.* In *P. bellina* (Fig. 3e) and *P. violacea* (Fig. 3g), *Orchid-rt1* repeats distributed in the intercalary regions of medium and large chromosomes and throughout all small chromosomes. According to the intensity of 4′, 6-diamidino-2-phenylindole (DAPI) staining, the intercalary regions of the medium and large chromosomes were mainly euchromatin, flanked by conspicuous heterochromatin (Fig. 3f, h).

### Gene expression affected by *Orchid-rt1* insertion

It has been found that the *P. equestris* genome has high abundance (about 60%) of transposon elements (TEs). Unlike other plants, these TEs are enriched in the intron regions [11]. Recently, we found that gene expression of *PeMYB11* is highly upregulated by the insertion of a retrotransposon, *HORT1* and results dark flower color in harlequin *Phalaenopsis* [22]. We wondered whether the insertion of *Orchid-rt1* may affect gene expressions*.* For this, whole genome alignment was performed by using the full length of *Orchid-rt1* against the *P. equestris* genome sequence. Among them, 38 of 43 hits were located on chromosomes, and 5 hits were located on unassembled scaffolds. In addition, 7 of 38 hits were inserted within genes, thus their paralogous genes were also identified. We then compared the gene expression levels among various organs of the 7 genes inserted with *Orchid-rt1* and their paralogs from the RNAseq data in OrchidBase (http://orchidbase.itps.ncku.edu.tw).

To confirm whether these genes do contain portions of *Orchid-rt1* sequence, primers around the predicted *Orchid-rt1* insertion sites were designed from three predicted *Orchid-rt1* inserted genes, *Peq018599*, *Peq009948*, and *Peq014239*. The amplified regions were sequenced and aligned to the *Orchid-rt1* sequence. A 600-bp fragment from *Orchid-rt1* was found within the predicted insertion region from *Peq009948* (Additional file 3: Fig. S2), but only short and separated matched fragments for the other two genes. This suggests that some predicted gene do harbor the *Orchid-rt1* region.

**Fig. 4:**
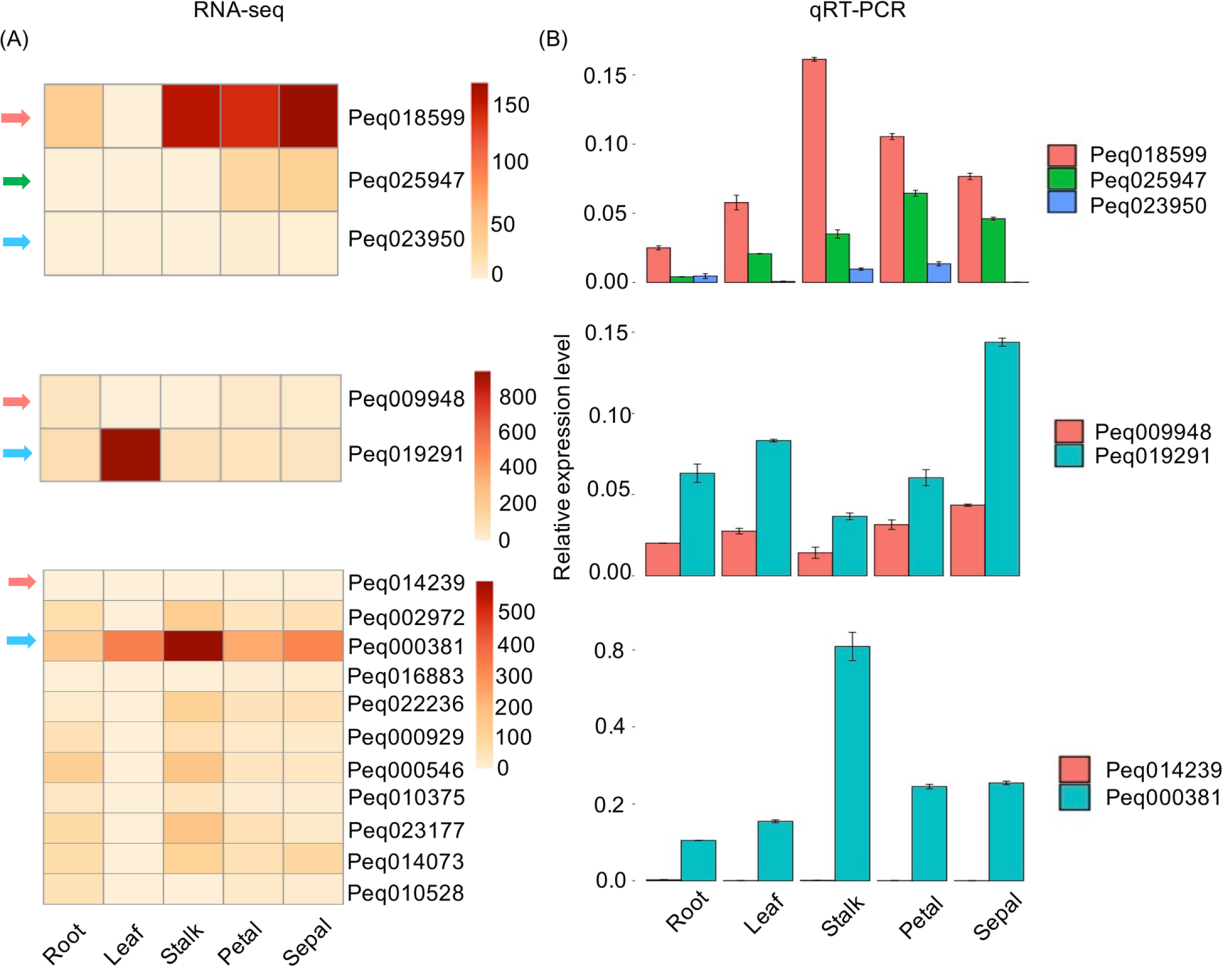
Gene expression from three TE predicted insertion genes and their paralogs. **a** Gene expression level of predicted TE inserted gene *Peq018599*, *Peq009948, Peq014239* and their paralogs among 5 different organs from RNAseq. The orange arrow indicates the *Orchid-rt1* partially inserted genes, green and blue arrows displayed the paralogous genes. **b** Validation of the gene expression by using qRT-PCR in triplicates, and repeated in three bio-replicates independently

Among these 7 genes, expression levels of one gene (*Peq018599*) is highly upregulated in all organs as compared to that of its paralogous genes, while expression levels of 2 genes (*Peq009948* and *Peq014239*) are down-regulated as compared to that of their paralogous genes (Fig. 4a). qRT-PCR was applied to validate the expression level of the predicted *Orchid-rt1* inserted genes and its paralogous genes (Fig. 4b). The predicted *Orchid-rt1* inserted gene *Peq018599* showed up-regulated expression as compared to its paralogous genes *Peq025947* and *Peq023950* in RNA-seq data and confirmed with qRT-PCR. In contrast, the other two predicted *Orchid-rt1* inserted genes *Peq009948* and *Peq014239* showed down-regulated expression compared to their paralogous genes *Peq019291* and *Peq000381*, respectively, in RNA-seq analysis, and this was confirmed by using qRT-PCR (Fig. 4b). This indicates that there might be association between the TE insertion and gene expression.

**Fig. 5:**
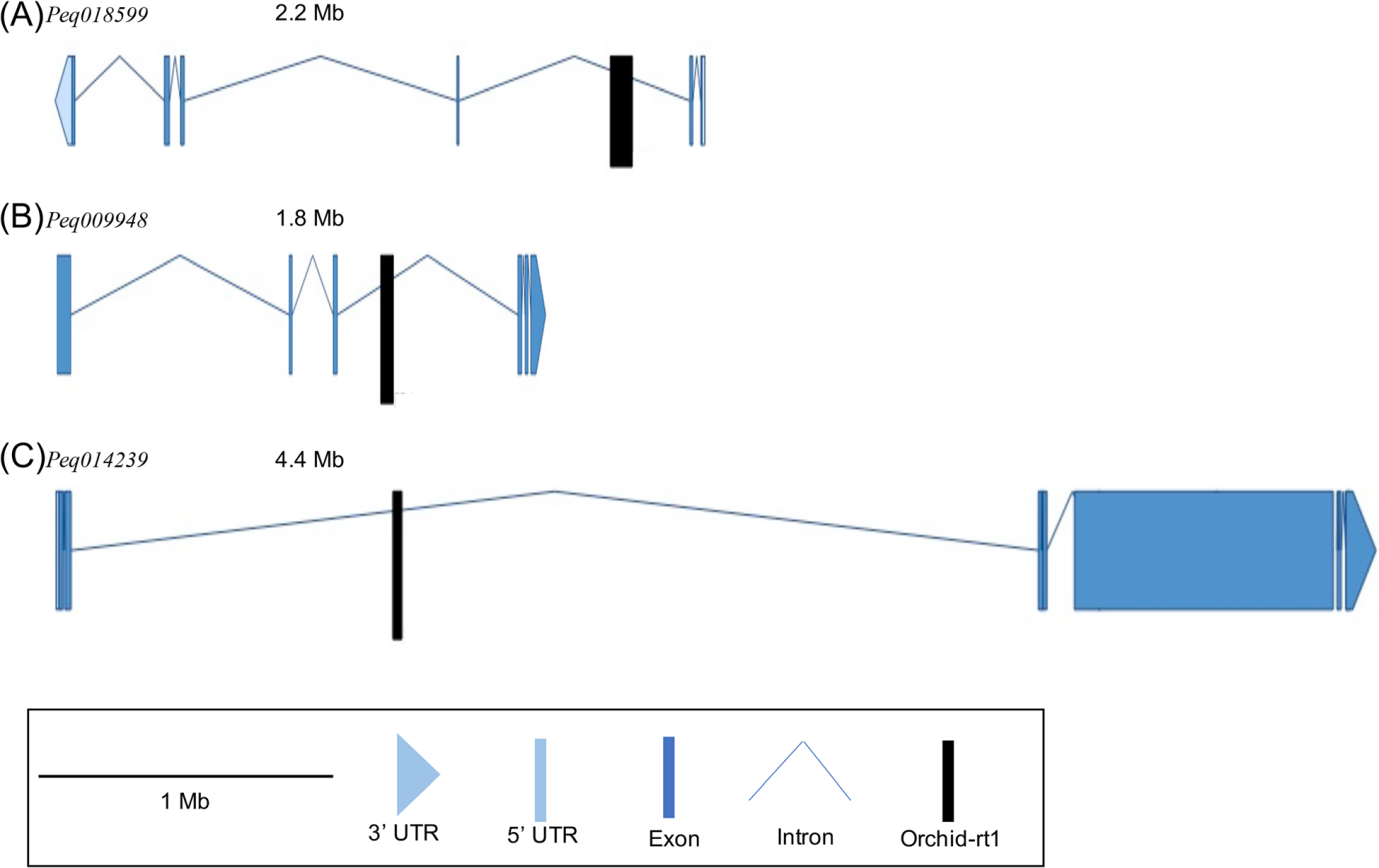
Insertion site of *Orchid-rt1* in the three genes with their gene expression levels been affected with the TE insertion. **a** *Peq018599*, putative function of Copper transporter 1; (**b**) *Peq009948*, predicted function of 26S proteasome non-ATPase regulatory subunit 4 homolog isoform 1; (**c**) *Peq014239*, annotated function of transcriptional factor B3 family protein

Expression levels of four other genes were not significantly affected with the insertion of *Orchid-rt1* as compared to their paralogs (Additional file 4: Table S2). Intriguingly, the above three genes (*Peq018599*, *Peq009948*, and *Peq014239*) with their expression levels been traumatized all with the insertion of *Orchid-rt1* in the intron regions with various size of 765 bp, 473 bp and 297 bp, respectively (Fig. 5).

## Discussion

In this study, we identified several LTR retrotransposons from *P. equestris* genome sequences for full-length and high-copy elements and characterized their amount and distribution pattern in four *Phalaenopsis* genomes. Among them, four *Gypsy*-like LTR retrotransposons, *Orchid-rt1*, *Gypsy1*, *Gypsy2*, and *Gypsy3*, represented a large amount of the *Phalaenopsis* genomes, while the increased copies of both *Orchid-rt1* and *Gypsy1* in two species with large genome size/chromosome size, *P. violacea* and *P. bellina*, suggest that these two retrotransposons are associated with genome size expansion. Based on a big comparative study for eight high-quality genomes, including *Arabidopsis thaliana*, *A. lyrata*, *Brachypodium dystachion*, grapevine (*Vitis vinifera*), maize (*Zea mays*), rice (*Oryza sativa*), sorghum (*Sorghum bicolor*), and soybean (*Glycine max*), it is a clear correlation between genome size to the retrotranspositional activity of the few retrotransposon families with most highly repeated, but not to a high number of repeated families [30]. Moreover, only one or few LTR retrotransposons were active recently in each genome, and the latest retrotranspositional burst(s) mostly were accounted for the difference in plant genome sizes [30]. Therefore, it is necessary to identify the LTR retrotransposons and investigate their effects on each plant genome.

Two approaches have been performed for the identification of the high-copy LTR retrotransposons from the *Phalaenopsis* genome, including LTRfinder for the full-length LTR retrotransposons from the assembled sequences, and the prediction of high-copy retrotransposons with high similarity from the most sequenced reads in the sequenced raw data. The prediction of LTRfinder for the full-length LTR retrotransposons with the protein-coding region for retrotransposition bordered by two identical LTRs was performed in several plant genomes, such as cassava (*Manihot esculenta*) [31], citrus (*Citrus x clementina*) [32], cotton (*Gossypium hirsutum*) [33], flax (*Linum usitatissimum* L.) [34], *Medicago truncatula* [35], and wheat (*Triticum aestivum*) [36]. Therefore, the 906 *Gypsy-*like and 402 *Copia*-like retrotransposons identified in this study were the predicted full-length retrotransposons with the protein-coding region bordered by two identical LTRs.

In addition, considering the composition of LTR retrotransposons is originated from the copy-and-paste strategy, the reads from the sequenced raw data will be assembled into a single contig among the draft genome sequence based on the sequence similarity. To avoid this, the second approach was performed via the prediction of high-copy retrotransposons with high similarities from the most sequenced reads in the sequenced raw data. Therefore, *Orchid-rt1* has been identified and predicted to be present with 18,000 copies in the *P. equestris* genome, but only 43 hits could be identified from the assembled whole genome sequences. The fact of high-copy numbers of *Orchid-rt1* present in *Phalaenopsis* genomes was then confirmed by using Southern blot, slot blot, and FISH results. It indicated that each copy of *Orchid-rt1* was similar to each other, while the amplified copies of *Orchid-rt1* were recently occurred without many mutations accumulated. As a fate of the retrotransposition event for LTR retrotransposons, the sequences of the newly inserted copy are identical to the original one, while mutations accumulated and resulting sequence divergence of these two copies over time, whose extent is proportional to the time elapsed since the insertion [15].

FISH results showed *Orchid-rt1* dispersed throughout all chromosomes in *P. equestris* and *P. aphrodite*, while only in the intercalary regions of medium and large chromosomes and throughout all small chromosomes in *P. bellina* and *P. violacea*. Considering that the studies of genome size variation among *Phalaenopsis* species show a positive relation between genome size and the amount of constitutive heterochromatin, the differential accumulation of heterochromatin is considered a major cause of karyotype variation in *Phalaenopsis* orchids [5]. Therefore, FISH results showed amplification of *Orchid-rt1* in the euchromatin regions also contributing to the expanded genome sizes. Similar situation was also found in the cytogenetic analysis on *Brachiaria* genus, while an *Athila* retrotransposon was detected mainly in the centromeric-pericentromeric regions of chromosomes of diploids *B. decumbens*, *B. ruziziensis*, and *B. brizantha*, but differences in location and signal strength in the polyploid *B. brizantha* [37].

Expression levels of genes inserted with *Orchid-rt1* in *P. equestris* genome are upregulated, downregulated, or non-significantly affected. We found 3 genes that have their expression levels regulated with the *Orchid-rt1* insertion. *Peq018599* encoding for copper transporter 1 with the insertion of *Orchid-rt1* showed highly upregulated gene expression in root and seed development as compared to its paralogs. Copper transporter 1 plays an important role in symbiotic nitrogen fixation in *Medicago truncatula* [38], functions in root elongation and pollen development in Arabidopsis [39], and involves in the copper transport in root of grapevine [40]. Two genes, *Peq009948* and *Peq014239* encoding for 26S proteasome non-ATPase regulatory subunit 4 homolog isoform 1 and transcriptional factor B3 family protein/ auxin-responsive factor AUX/IAA-related, respectively, were down-regulated in all organs and seed development as compared to their paralogs. The 26S proteasome is a complex in charge of protein turn over. The 26S proteasome contains one 20S core particle and two 19S regulatory particles (RPs). The RPN12a protein, a non-ATPase subunit of the 19S RP, is involved in cytokinin signaling in *Arabidopsis* [41]. Expression level of *Peq009948* is reduced to half amount of its paralog in most organs, and abolished in the floral stalk and leaf. Auxin response factor is a member of the plant-specific B3 DNA binding superfamily [42]. In the *Peq014239*, its expression is almost abolished in the floral stalk, leaf and root, trace amount in sepal and seed development, and a substantial amount in petal, labellum, pollinium and gynostemium. All these three genes are involved in growth and development, and further analysis will be required to detail the significance of regulation of gene expression upon the insertion of *Orchid-rt1* into these genes.

## Conclusion

In this study, we identified several LTR retrotransposons from *P. equestris* genome sequences for full-length and high-copy elements and characterized their amount and distribution pattern in four *Phalaenopsis* genomes. Four *Gypsy*-like LTR retrotransposons, *Orchid-rt1*, *Gypsy1*, *Gypsy2*, and *Gypsy3*, represented a large amount of the *Phalaenopsis* genomes. Both *Orchid-rt1* and *Gypsy1* showed increased copies in two species with large genome size/chromosome size, *P. violacea* and *P. bellina*, which suggests that these two retrotransposons may be associated with genome size expansion. FISH results revealed that abundant *Orchid-rt1* mainly distributed in the intercalary euchromatin regions of large chromosomes in these two species. Our results support that these four retrotransposons amplified themselves within *Phalaenopsis* genomes, accompanied by expanded genome sizes. Insertion of *Orchid-rt1* is found in the intron regions and gene expression are affected upon the insertion of *Orchid-rt1.*

## Methods

### Plant materials

We tested four native *Phalaenopsis* orchids with small genome size/small chromosomes or large genome size/large chromosomes accompanied by small chromosomes. *P. aphrodite* and *P. equestris* have small genomes, and *P. bellina* and *P. violacea* have large genomes**.** All plant materials were grown in the greenhouse at National Cheng Kung University (Tainan, Taiwan) under natural light and controlled temperature from 23 °C to 27 °C.

### Sequencing the *Phalaenopsis* genomic DNA

The analyzed sequences were obtained from the previously sequenced 21,350 BAC end sequences (BESs), and the sequenced *Phalaenopsis* genome by using an NGS strategy with the Illumina/Solexa platform. The genomic DNA was extracted from leaves of *P. equestris*, sheared into 40-kb sequences by using HydroShear (Digilab, Marlborough, MA, USA), and sequenced by using Roche 454 and Illumina/Solexa for one run. The sequences from the BESs, Roche 454, and Illumina/Solexa data were assembled into large contigs by using Sequencher 4.2 (Gene Codes Corp., Ann Arbor, MI).

### Identification and characterization of TEs in *P. equestris* genome

The assembled large contigs were used to search for TEs with on-line TE-prediction software, LTRfinder, LTRharvest, and RepeatMasker. LTRfinder and LTRharvest are software tools for automated annotation of internal features of putative LTR retrotransposons. RepeatMasker is used to screen DNA sequences for interspersed repeats and low-complexity DNA sequences. Each predicted TE was verified for its terminal repeats on both ends and identified for other copies in the *Phalaenopsis* genome by using NCBI-BLAST2 Sequences (http://www.ncbi.nlm.nih.gov/ blast/bl2seq/wblast2.cgi/) and Gepard, a rapid and sensitive tool for creating dotplots on a genome scale [25]. NCBI-BLAST2 Sequences and Gepard provide easy and powerful means of sequence analysis, useful for searching repeats within a single sequence and regions of similarity in two sequences. The TEs with their similar copies were grouped into the same families. The probable protein-coding regions among the terminal repeat sequences were predicted by using BLASTX with the NCBI non-redundant (nr) protein sequences database. The length of LTR sequences for each predicted LTR retrotransposon were analyzed by using NCBI-BLAST2 Sequences and multiple alignment software. The autonomous elements and the complete one for each TE family were retained for finding the other copies for each TE by using BLASTN. Each TE family was identified and characterized with its sequence structure and copy number in the *Phalaenopsis* genome. Moreover, each characterized TE was analyzed for their contribution to *Phalaenopsis* genome structure.

### Slot blot hybridization

Genomic DNAs were isolated from flowers of *P. aphrodite*, *P. equestris*, *P. bellina* and *P. violacea.* Slot blot assay was performed with 1 μg genomic DNA, 10 ng plasmid DNA, and a serial dilution of DNAs. These DNAs were loaded onto nylon filters by using Hoefer PR648 (Hoefer Inc., Holliston, MA, USA). Probes were amplified from the genomic DNA of *P. equestris* for the LTR sequences for *Orchid-rt1*, *Gypsy1*, *Gypsy2*, and *Gypsy3* and the open reading frame sequences for *Orchid-rt1* and *Gypsy1*. Slot blot hybridization followed the standard protocol [43] with the AlkPhos Direct Labelling and Detection System and the CDP-Star kit (Amersham Pharmacia Biotech).

### Fluorescence in situ hybridization (FISH) analysis

Preparation of *P. equestris*, *P. aphrodite*, *P. violacea*, and *P. bellina* chromosome spreads and FISH procedures followed dual-color FISH protocols [44]. Briefly, root tips were treated with 2 mM 8-hyroxyquinoline, fixed with Farmer’s solution, and macerated with 2% cellulose (Onozuka R-10, Yakult Honsha, Tokyo) and 2% pectinase (Sigma Chemical Co.) in 10 mM citrate buffer, pH 4.0, at 37 °C for 90 min, then squashed on slides in the same fixative. The probe for the LTR sequence for *Orchid-rt1* was labeled with digoxigenin-11-dUTP (Roche Diagnostics) via nick translation according to a standard protocol. Post-hybridization washing, signal detection, and image processing were performed as described [44]. Chromosomes were counterstained with 4′, 6-diamidino-2-phenylindole (DAPI) in an antifade solution (Vector Laboratories, CA, USA). FISH images were recorded by use of an epifluorescence microscope (AxioImager A1, Carl Zeiss AG, Jena, Germany) equipped with a cooled CCD camera (AxioCam MRm). Figures were edited by using Adobe Photoshop 9.0 (Adobe Systems Inc., USA). For each accession, at least five chromosome complements with good labelling signals were photographed and analyzed.

### Expression levels of genes inserted by *Orchid-rt1* and their paralogs

To find out the genes that have insertions of *Orchid-rt1*, whole genome alignment was performed by using the full length of *Orchid-rt1* against the *P. equestris* genome sequence with the software LAST [45]. Among them, 38 of 43 hits were located on chromosomes, and the others were located on unassembled scaffolds. Furthermore, 7 of 38 were participated within the genes. The paralog genes of above 7 genes were identified by using the full CDS for tblastx in Orchidbase database (http://orchidbase.itps.ncku.edu.tw/ est/home2012.aspx) based on the threshold that score higher than 100 and E value lower than 0.00001. To confirm the insertion of *Orchid-rt1* in the predicted genes, primers were design from outside of predicted insertion sites by primer3plus (http://www.bioinformatics.nl/cgi-bin/primer3plus/ primer3plus.cgi/). The amplified fragments were sequenced and BLAST against *Orchid-rt1* full-length sequence using the global alignment in NCBI (https://blast.ncbi.nlm.nih.gov/Blast.cgi).

The relative gene expression levels of those genes inserted with *Orchid-rt1* and their paralogous genes in several organs were found in the Orchidbase as well and presented as heatmap. The up-regulated or down-regulated gene expression of the inserted genes compared to their paralogs identified in the RAN-seq data were further confirmed by using qRT-PCR. The cDNA samples were prepared from 5 different organs including root, leaf, stalk, petal and sepal. Were mixed with 2X SYBR Green PCR master mix (Applied Biosystems, Norwalk, CT, USA) and analyzed in an ABI 7300 instrument (Applied Biosystems). Each sample was performed in triplicates as technical repeats with the following reactions: Incubation at 50 °C for 2 min, then 95 °C for 10 min, and thermal cycling for 40 cycles (95 °C for 15 s and 60 °C for 1 min). The relative expression level was calculated according to the manufacturer’s instructions (Applied Biosystems). qRT-PCR was performed with three different plants as bio-replicates. The *Peactin1* was applied as internal control for normalization.

## Supplementary Information

**Additional file 1:** **Figure S1.** Four native *Phalaenopsis* species analyzed in the study, including *P. aphrodite* subsp. *formosana* (A) and *P. equestris* (B) with small genome/small chromosomes, and *P. bellina* (C) and *P. violacea* (D) with large genome/large chromosomes.

**Additional file 2:** **Table S1.** The copy numbers of the predicted *Copia*-like retrotransposons.

**Additional file 3:** **Figure S2.** The global alignment result between *Orchid-rt1* full length and predicted insertion region from *Peq009948*.

**Additional file 4:** **Table S2.** The expression levels of *Orchid-rt1* inserted gene and their paralogs genes among different organs.

## Data Availability

Original sequences of the BESs, Roche 454, Illumina/Solexa data, and the identified LTR retrotransposons can be accessed at Orchidbase database (http://orchidbase.itps.ncku.edu.tw/est/home2012.aspx).

## Abbreviations

BES: BAC end sequences
CDS: Coding sequence
FISH: Fluorescence in situ hybridization
LTRs: Long Terminal Repeats
RPs: Regulatory particles
TEs: Transposable elements
