# Supplementary material for: Identification of high-copy number long terminal repeat retrotransposons and their expansion in Phalaenopsis orchids
Source: BMC Genomics. 2020 Nov 19;21:807. doi: 10.1186/s12864-020-07221-6 (PMC7678294; doi:10.1186/s12864-020-07221-6)
Supplement: Supplementary file 1 — Additional file 1: Figure S1. Four native Phalaenopsis species analyzed in the study, including P. aphrodite subsp. formosana (A) and P. equestris (B) with small genome/small chromosomes, and P. bellina (C) and P. violacea (D) with large genome/large chromosomes. [file 12864_2020_7221_MOESM1_ESM.docx]

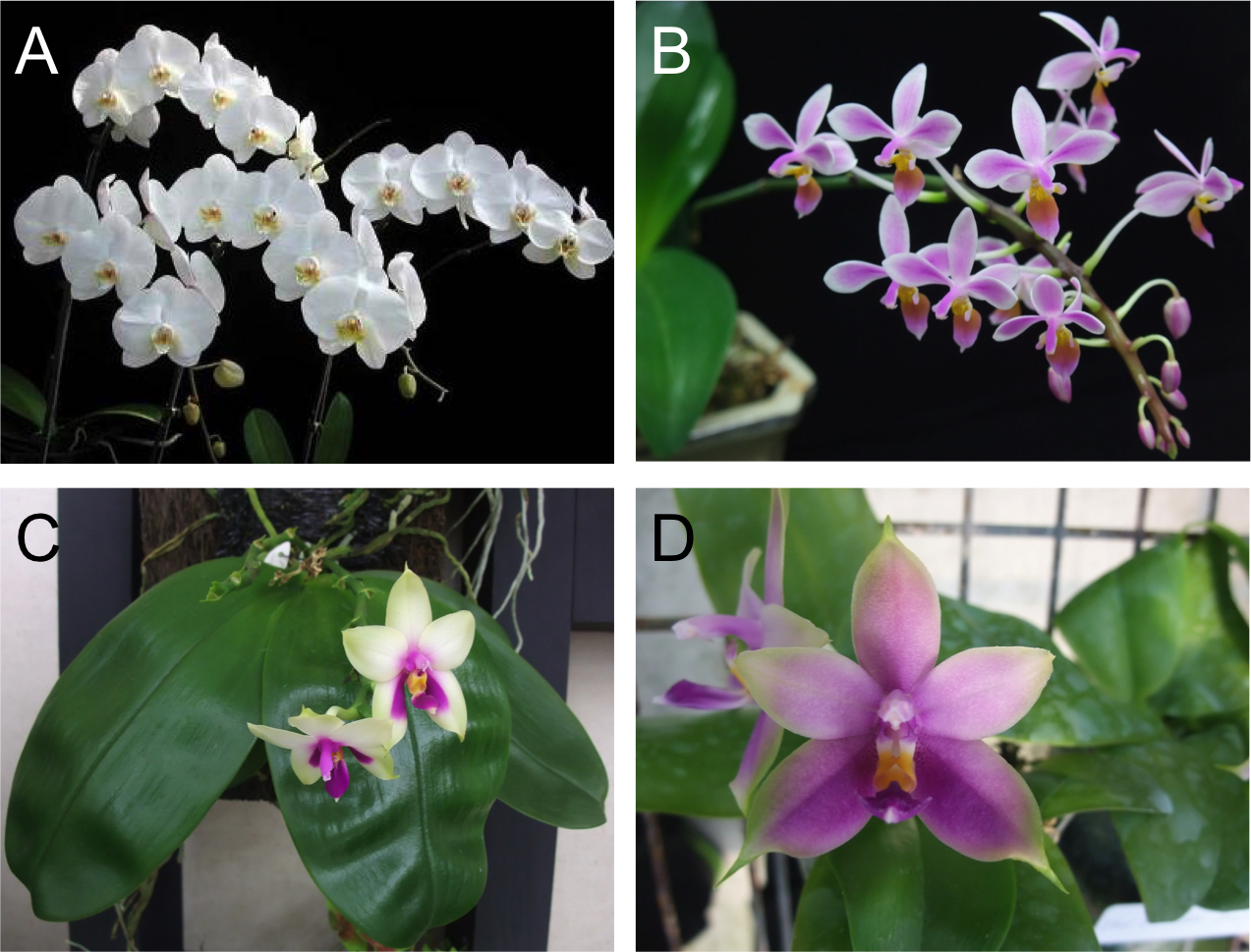


**Additional file 1: Fig. S1.** Four native *Phalaenopsis* species analyzed in the study, including *P. aphrodite* subsp. *formosana* (A) and *P. equestris* (B) with small genome/small chromosomes, and *P. bellina* (C) and *P. violacea* (D) with large genome/large chromosomes.
