# Supplementary material for: Identification of high-copy number long terminal repeat retrotransposons and their expansion in Phalaenopsis orchids
Source: BMC Genomics. 2020 Nov 19;21:807. doi: 10.1186/s12864-020-07221-6 (PMC7678294; doi:10.1186/s12864-020-07221-6)
Supplement: Supplementary file 2 — Additional file 2: Table S1. The copy numbers of the predicted Copia-like retrotransposons. [file 12864_2020_7221_MOESM2_ESM.docx]

**Additional file 2: Table S1.** The copy numbers of the predicted *Copia*-like retrotransposons

| Family name | No. of elements | Total length (bp) | LTR length | CDS length (bp) |
| --- | --- | --- | --- | --- |
| *Copia1* | 48 | 5260 | 238 | 4340 |
| *Copia2* | 23 | 4940 | 270 | 3650 |
| *Copia3* | 21 | 4609 | 331 | 1041 |
| *Copia4* | 19 | 5555 | 360 | 3815 |
| *Copia5* | 19 | 6443 | 685 | 3650 |
| *Copia6* | 19 | 5451 | 625 | 3931 |
| *Copia7* | 16 | 5339 | 242 | 2240 |
| *Copia8* | 14 | 4912 | 379 | 3612 |
| *Copia9* | 14 | 5393 | 648 | 1440 |
| *Copia10* | 13 | 2869 | 545 | 1495 |
| *Copia11* | 9 | 1723 | 550 | 280 |
| *Copia12* | 9 | 2734 | 274 | 1304 |
| *Copia13* | 8 | 5532 | 309 | 4799 |
| *Copia14* | 8 | 6261 | 425 | 3960 |
| *Copia15* | 6 | 4341 | 252 | 1292 |
| *Copia16* | 6 | 6207 | 523 | 3874 |
| *Copia17* | 6 | 4918 | 182 | 4148 |
| *Copia18* | 5 | 5334 | 436 | 2402 |
| *Copia19* | 4 | 5226 | 256 | 3960 |
| *Copia20* | 2 | 6136 | 1181 | 438 |
| Singleton | 115 |  |  |  |
| total | 402 |  |  |  |
