# Supplementary material for: Identification of high-copy number long terminal repeat retrotransposons and their expansion in Phalaenopsis orchids
Source: BMC Genomics. 2020 Nov 19;21:807. doi: 10.1186/s12864-020-07221-6 (PMC7678294; doi:10.1186/s12864-020-07221-6)

**Additional file 3: Fig. S2**. The global alignment result between *Orchid-rt1* full length and predicted insertion region from *Peq009948*. “Query” indicates the full length of *Orchid-rt1*, the “Sbjct” represents the predicted insertion region of *Peq009948*.


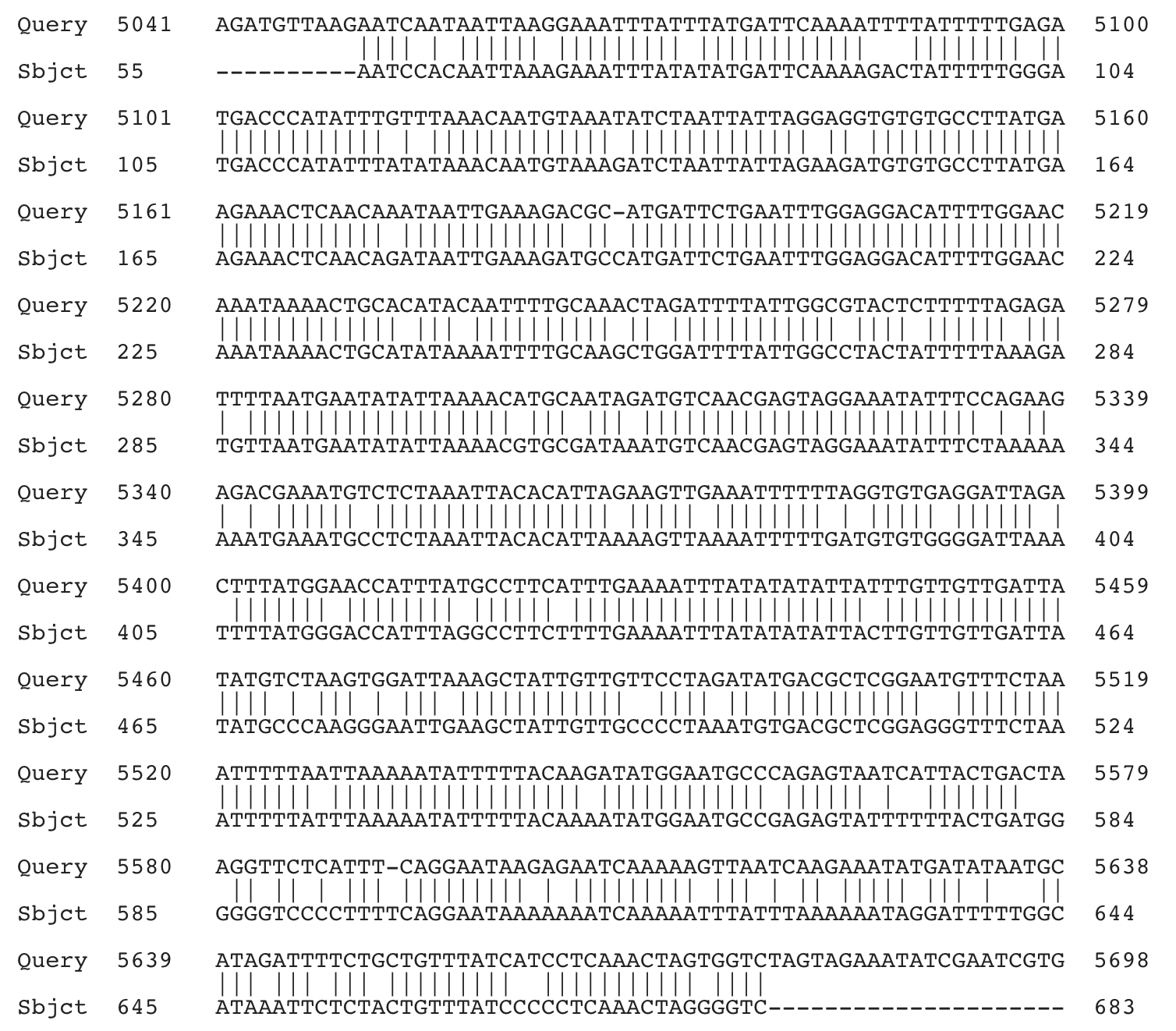

Supplement: Supplementary file 3 — Additional file 3: Figure S2. The global alignment result between Orchid-rt1 full length and predicted insertion region from Peq009948. [file 12864_2020_7221_MOESM3_ESM.docx]
