# Supplementary material for: Identification of high-copy number long terminal repeat retrotransposons and their expansion in Phalaenopsis orchids
Source: BMC Genomics. 2020 Nov 19;21:807. doi: 10.1186/s12864-020-07221-6 (PMC7678294; doi:10.1186/s12864-020-07221-6)
Supplement: Supplementary file 4 — Additional file 4: Table S2. The expression levels of Orchid-rt1 inserted gene and their paralogs genes among different organs. [file 12864_2020_7221_MOESM4_ESM.docx]

**Additional file 4: Table S2.** The expression levels of *Orchid-rt1* inserted gene and their paralogs genes among different organs.

| Type | [Gene ID](javascript:__doPostBack('gv','Sort$geneid')) | [Sepal](javascript:__doPostBack('gv','Sort$Sepal')) | [Petal](javascript:__doPostBack('gv','Sort$Petal')) | [Labellum](javascript:__doPostBack('gv','Sort$Labellum')) | [Pollinium](javascript:__doPostBack('gv','Sort$Pollinium')) | [Gynostemium](javascript:__doPostBack('gv','Sort$Gynostemium')) | [Floral stalk](javascript:__doPostBack('gv','Sort$Floral%20stalk')) | [Leaf](javascript:__doPostBack('gv','Sort$Leaf')) | [Root](javascript:__doPostBack('gv','Sort$Root')) | [4 Day Seed](javascript:__doPostBack('gv','Sort$4%20Day%20Seed')) | [7 Day Seed](javascript:__doPostBack('gv','Sort$7%20Day%20Seed')) | [12 Day Seed](javascript:__doPostBack('gv','Sort$12%20Day%20Seed')) |
| --- | --- | --- | --- | --- | --- | --- | --- | --- | --- | --- | --- | --- |
| **Inserted^a^** | ***Peq018599*** | **169.97^b^** | **144.08** | **131.55** | **71.21** | **123.53** | **155.84** | **0** | **36.52** | **112.59** | **125.98** | **117.14** |
| Paralog | *Peq025947* | 31.13 | 26.36 | 54.82 | 4.93 | 28.07 | 0 | 0 | 0 | 1.81 | 3.13 | 1.22 |
| Paralog | *Peq023950* | 1.6 | 1.98 | 0.25 | 0.98 | 4.39 | 0 | 0 | 0.63 | 0.11 | 0 | 0 |
| **inserted** | ***Peq009948*** | **25.26** | **36.84** | **35.83** | **44.72** | **25** | **0** | **0** | **55.21** | **66.4** | **46.87** | **46.55** |
| Paralog | *Peq019291* | 59.35 | 66.56 | 54.02 | 33.47 | 46.49 | 76.93 | 942.67 | 99.65 | 81.37 | 68.97 | 74.14 |
| **inserted** | ***Peq014239*** | **0.62** | **2.79** | **8.63** | **4.93** | **6.98** | **0** | **0** | **0** | **0.16** | **0.27** | **0.41** |
| Paralog | *Peq002972* | 55.73 | 39.01 | 23.38 | 9.55 | 33.76 | 129.09 | 0 | 67.56 | 93.48 | 73.15 | 53.15 |
| Paralog | *Peq000381* | 315.27 | 236.05 | 251.95 | 19.19 | 191.62 | 597.6 | 332.83 | 141.65 | 62.47 | 53.29 | 67.17 |
| Paralog | *Peq016883* | 14.21 | 11.06 | 18.63 | 9.42 | 13.5 | 0 | 0 | 3.22 | 4.58 | 5.42 | 6.54 |
| Paralog | *Peq022236* | 55.28 | 46.68 | 47.54 | 4.6 | 32.76 | 109.35 | 0 | 18.79 | 12.06 | 11.02 | 13.58 |
| Paralog | *Peq000929* | 23.92 | 26.62 | 47.18 | 21.31 | 32.88 | 58.09 | 0 | 54.33 | 10.02 | 9.58 | 8.96 |
| Paralog | *Peq000546* | 33.93 | 38.74 | 48.42 | 38.16 | 50.73 | 162.08 | 0 | 122.8 | 20.86 | 21.75 | 26.6 |
| Paralog | *Peq010375* | 12.52 | 11.7 | 25.23 | 5.95 | 15.4 | 38.17 | 0 | 32.68 | 42.41 | 46.86 | 61.95 |
| Paralog | *Peq023177* | 22.67 | 54.99 | 50.82 | 55.86 | 112.66 | 169.3 | 0 | 81.36 | 93.85 | 108.2 | 104.06 |
| Paralog | *Peq014073* | 89.24 | 57.26 | 71.88 | 26.08 | 68.9 | 103.23 | 0 | 68.17 | 33.38 | 30.94 | 33.92 |
| Paralog | *Peq010528* | 14.38 | 22.41 | 44.92 | 14.19 | 35.79 | 0 | 0 | 52.36 | 20.03 | 22.01 | 19.93 |
| **inserted** | ***Peq010304*** | **9.68** | **3.31** | **13.3** | **4.98** | **4.98** | **0** | **0** | **0.63** | **44.87** | **50.89** | **46.7** |
| Paralog | *Peq019211* | 6.09 | 8.56 | 10.45 | 0.18 | 7.44 | 0 | 0 | 1.4 | 70.64 | 79.98 | 60.46 |
| Paralog | *Peq014291* | 11.74 | 2.47 | 5.31 | 31.78 | 9.61 | 0 | 0 | 1.38 | 7.29 | 9.97 | 12.71 |
| **inserted** | ***Peq010258*** | **7.06** | **8.3** | **10.43** | **1.91** | **9.15** | **0** | **0** | **9.63** | **4.72** | **4.8** | **2.74** |
| Paralog | *Peq025188* | 6.45 | 4.3 | 4.4 | 3.9 | 5.24 | 34.48 | 0 | 5.58 | 2.87 | 1.68 | 2.13 |
| Paralog | *Peq015287* | 5.4 | 3.99 | 4.78 | 2.39 | 3.36 | 34.48 | 0 | 4.11 | 1.41 | 0.79 | 1.53 |
| Paralog | *Peq006658* | 13.54 | 11.75 | 11.22 | 7.67 | 12.03 | 0 | 0 | 19.65 | 13.46 | 11.82 | 11.43 |
| Paralog | *Peq019563* | 12.37 | 8.43 | 11.15 | 7.68 | 10.35 | 0 | 0 | 18.47 | 8.17 | 6.33 | 10.04 |
| Paralog | *Peq004701* | 11.11 | 12.09 | 12.56 | 10.27 | 18.48 | 103.16 | 0 | 41.31 | 19.45 | 18.39 | 16.66 |
| Paralog | *Peq021618* | 8.93 | 10.76 | 11.76 | 6 | 13.09 | 38.47 | 0 | 5.24 | 8.95 | 10.32 | 8.38 |
| Paralog | *Peq021104* | 15.93 | 18.6 | 18.01 | 19.33 | 21.22 | 0 | 0 | 57.27 | 12.32 | 10.46 | 9.06 |
| Paralog | *Peq015006* | 5.76 | 6.82 | 7.69 | 4.68 | 8.98 | 0 | 0 | 8.39 | 6.22 | 6.1 | 4.46 |
| Paralog | *Peq023403* | 6.84 | 5.66 | 5.72 | 13.11 | 9.13 | 29.12 | 0 | 14.02 | 11.25 | 10.15 | 10.32 |
| Paralog | *Peq011489* | 14.27 | 20.58 | 16.12 | 9.91 | 17.83 | 0 | 0 | 23.51 | 24.48 | 24.47 | 18.86 |
| Paralog | *Peq003141* | 6.74 | 8.4 | 6.86 | 16.13 | 14.14 | 0 | 0 | 17.05 | 6.88 | 8.32 | 7.21 |
| Paralog | *Peq009114* | 8.68 | 11.72 | 9.29 | 26.22 | 17.65 | 0 | 0 | 17.2 | 15.4 | 12.22 | 14.31 |
| Paralog | *Peq006249* | 7.5 | 8.34 | 6.64 | 5.67 | 7.2 | 0 | 0 | 10.82 | 5.72 | 5.66 | 6.23 |
| Paralog | *Peq022478* | 12.07 | 10.16 | 8.7 | 27.5 | 16.92 | 56.02 | 0 | 36.52 | 20.46 | 20.07 | 23.31 |
| Paralog | *Peq015628* | 11.31 | 15.68 | 12.66 | 13.48 | 13.98 | 0 | 0 | 13.07 | 12.9 | 12.05 | 10.02 |
| Paralog | *Peq002191* | 15.37 | 17.18 | 19.92 | 22.25 | 17.36 | 29.61 | 0 | 24.1 | 16.49 | 16.42 | 14.88 |
| Paralog | *Peq006747* | 15.49 | 13.34 | 15.31 | 45.55 | 18.78 | 37.06 | 0 | 41.21 | 15.07 | 15.96 | 15.26 |
| Paralog | *Peq004146* | 4.22 | 5.44 | 6.09 | 14.4 | 9.96 | 0 | 0 | 3.82 | 3.89 | 3.15 | 3.21 |
| Paralog | *Peq012532* | 20.47 | 21.63 | 27.09 | 42.49 | 25.18 | 0 | 0 | 25.99 | 32.46 | 40.17 | 28.9 |
| Paralog | *Peq005285* | 10.61 | 10.82 | 11.19 | 34.2 | 13.05 | 0 | 0 | 17.5 | 6.89 | 7.73 | 8.31 |
| Paralog | *Peq013284* | 8.78 | 8.23 | 8.1 | 6.46 | 7.35 | 35.44 | 0 | 3.93 | 11.41 | 12.36 | 9.81 |
| Paralog | *Peq015308* | 3.19 | 5.48 | 3.57 | 15.55 | 4.71 | 0 | 0 | 4.98 | 1.83 | 2.1 | 2.01 |
| Paralog | *Peq016196* | 0.18 | 0.61 | 0.23 | 0.2 | 0.62 | 0 | 0 | 0 | 0.21 | 0 | 0.27 |
| Paralog | *Peq008881* | 0 | 0 | 0 | 0.11 | 0 | 0 | 0 | 0 | 0 | 0 | 0 |
| Paralog | *Peq010403* | 0.44 | 0.34 | 0.76 | 0.12 | 0.97 | 0 | 0 | 2.89 | 0.17 | 0 | 0.16 |
| **Inserted^c^** | ***Peq022050*** | **34.71** | **86.06** | **47.62** | **545.8** | **226.74** | **80.77** | **0** | **36.13** | **72.46** | **71.18** | **66.28** |
| **Inserted^c^** | ***Peq003274*** | **28.06** | **26.29** | **21.37** | **9.85** | **25.47** | **35.52** | **0** | **57.65** | **13.41** | **15.25** | **20.6** |

^a^Green color show the gene with the insertion of *Orchid-rt1*.

^b^Expression level is presented as fragments per kilobase of transcript per million mapped reads (FPKM).

^c^No paralogs identified.
